# The role of parvalbumin neurons in the evolution of skilled behaviours

**DOI:** 10.1371/journal.pbio.3001795

**Published:** 2022-09-21

**Authors:** Jon T. Sakata, Sarah C. Woolley

**Affiliations:** 1 Department of Biology, McGill University, Montreal, Canada; 2 Centre for Research in Brain, Language and Music, McGill University, Montreal, Canada

## Abstract

Understanding how animals display diverse and complex behaviours remains a central question in biology. A new study in *PLOS Biology* suggests that the emergence of clusters of parvalbumin neurons in the forebrain could reflect a convergent mechanism underlying the evolution of skilled behaviours in birds.

Whether it is watching the courtship display of a sage grouse on a nature documentary or the response of one’s cat to a squirrel outside the window, people around the world are captivated by the diversity of behaviours exhibited by different animals. Behaviours are generated by activity within specific neural circuits, and novel behaviours can arise by the emergence of new brain areas, pathways, or cell types [1]. However, novel behaviours can also arise through modifications of anatomical structure (e.g., via segregation or lamination), neuromodulatory inputs, gene expression, or excitatory and inhibitory drive within existing brain areas [1]. Distinguishing between these possibilities is a longstanding challenge, and across some distantly related species, behavioural evolution can rely on similar brain modifications, reflecting a common evolutionary solution to behavioural diversification.

One well-studied example of shared mechanisms underlying behavioural evolution deals with vocal communication in songbirds, parrots, and hummingbirds. Not only do these birds produce diverse and melodic vocalisations, but also their songs are learned during development in a manner that parallels how humans acquire speech [2,3]. It is hypothesised that vocal learning evolved independently in these 3 clades of birds, offering a powerful opportunity to reveal shared and distinct mechanisms underlying the evolution of similar phenotypes. Intriguingly, each of these groups of vocal-learning birds possess distinct clusters of parvalbumin (PV) neurons within forebrain pathways specialised for communication [4]. While the precise locations of PV neuron clusters vary across songbirds, parrots, and hummingbirds, they are observed in forebrain areas known to be important for song learning and have not been observed in bird species that do not engage in vocal learning. Moreover, PV is enriched in parts of the human motor cortex that regulate speech, suggesting that the emergence of forebrain clusters of PV neurons could represent a shared molecular mechanism underlying the evolution of vocal learning and performance across birds and mammals [5].

In this issue of *PLOS Biology*, Schuppe and colleagues [6] document the presence of PV neuron clusters in the forebrain of 3 woodpecker species. Woodpeckers are unique and famous for hammering their bills against wood to excavate cavities for food, to create nests, and to communicate with others during territorial or courtship interactions [7]. Intriguingly, despite that woodpeckers are not considered vocal learners, the PV neuron clusters discovered in woodpeckers were observed in similar parts of the brain as in vocal-learning birds. Schuppe and colleagues [6] also report that brain areas enriched in PV are active during social interactions that involve drumming but not during social interactions without drumming (e.g., territorial singing) and that individual variation in brain activity in these areas correlates with individual variation in the amount of drumming. Although brain activity during only a few behaviours was examined, their analyses suggest that forebrain areas enriched with PV could be devoted specifically to drumming. Interestingly, brain areas activated during drumming are adjacent to brain areas active during non-drumming behaviours like flying, suggesting that drumming areas evolved from brain areas regulating other types of motor behaviours; similar hypotheses have been proposed for the evolution of song control nuclei in songbirds, parrots, and hummingbirds [8]. These initial findings lay the groundwork for further investigations in woodpeckers, including discerning the degree to which PV neurons themselves are active during drumming, whether species variation in forebrain PV neurons covaries with species variation in drumming speed and duration [7](Fig 1) and how manipulations of PV neuron activity modulate the production or perception of drumming.

**Fig 1:**
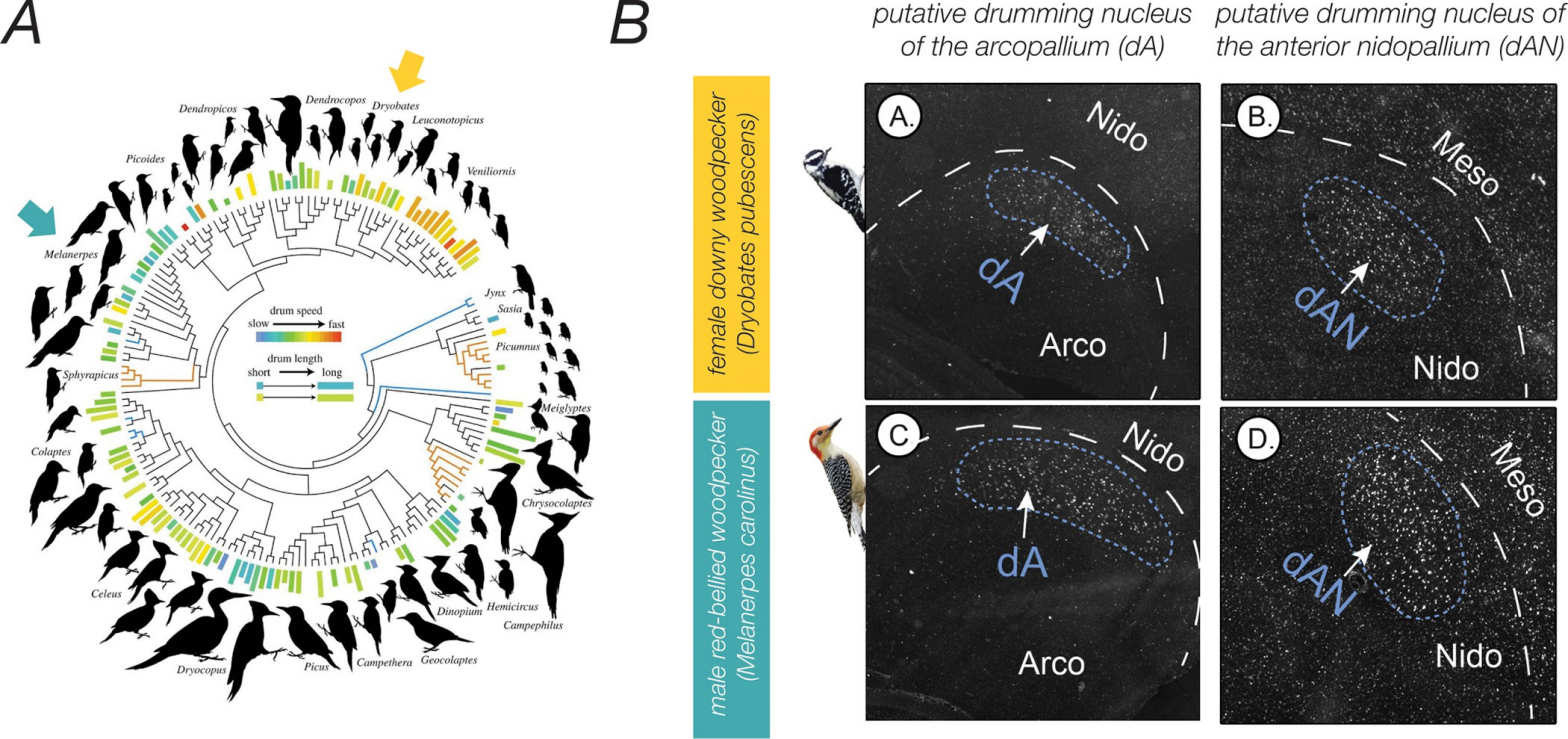
Drumming behaviour and expression of parvalbumin neurons in the forebrain of woodpeckers. (A) Phylogeny and species diversity of drumming in woodpeckers. Height of bars next to bird silhouettes summarises the duration of drums for individual species, and the colour of the bars indicates drumming speed for each species. Yellow and blue arrows highlight birds depicted in B. Adapted from [7]. (B) Clusters of parvalbumin neurons in 2 forebrain areas (the “dA” and “dAN”) of 2 woodpecker species. Adapted from [6].

Because the pattern of PV expression in woodpeckers resembles the convergent evolution of PV neurons in areas for vocal learning in birds [4,5] and because PV neurons regulate many forms of neural and behavioural plasticity [9,10], one of the primary questions raised by these findings is the role of learning in woodpecker drumming. Little is known about the degree to which rhythmic drumming is learned but, like learned vocalisations in vocal-learning species [3], the rhythmic drumming of woodpeckers involves a protracted period of development and is acutely modulated by social interactions. If drumming is indeed learned, it will be important to discern how PV neurons shape neural dynamics to allow for learning; for example, PV neurons in mammals are hypothesised to influence learning by modulating oscillatory patterns (e.g., gamma oscillations) and the balance of excitation and inhibition in focal circuits [9,10].

It will also be important to reveal how forebrain areas replete with PV neurons interface with each other and with other parts of the brain (e.g., the hindbrain and the spinal cord). Direct forebrain projections to hindbrain vocal motor regions are consistently observed in vocal-learning birds and mammals and are absent or sparse in species that do not learn their vocalisations [2]. Consequently, these direct projections are hypothesised to be critical to the evolution of vocal learning. In this respect, it will be important to characterise the extent to which neurons in a part of the avian brain called the arcopallium (Fig 1) directly project to hindbrain areas that regulate the muscles for drumming behaviour. Relatedly, specialised PV expression is also observed in hindbrain areas for vocal control in songbirds and humans [4,5], raising the question of specialisations in PV expression in the hindbrain of woodpeckers.

Finally, these findings broaden the discussion about the function of PV neurons in focal brain areas. While PV neurons in the forebrain of birds have been proposed to allow for behavioural plasticity, the emergence of PV neurons in similar parts of the brain (nidopallium and arcopallium) across woodpeckers and vocal-learning birds could be due to the fact that drumming in woodpeckers and song in songbirds, parrots, and hummingbirds are rapid, skilled behaviours involving the head. In this respect, the work by Schuppe and colleagues [6] motivates investigations into PV expression in similar parts of the brain among species that produce other types of skilled head movements as well as a general search for discrete PV populations in animals that display other types of complex, skilled behaviours including the choreographed dance displays of manakins.
